# Who is the research subject in cluster randomized trials in health research?

**DOI:** 10.1186/1745-6215-12-183

**Published:** 2011-07-26

**Authors:** Andrew D McRae, Charles Weijer, Ariella Binik, Angela White, Jeremy M Grimshaw, Robert Boruch, Jamie C Brehaut, Allan Donner, Martin P Eccles, Raphael Saginur, Merrick Zwarenstein, Monica Taljaard

**Affiliations:** 1 Department of Epidemiology and Biostatistics, University of Western Ontario, London, ON, N6A 5C1, Canada; 2 Division of Emergency Medicine, University of Calgary, Foothills Medical Centre, 1403 29th Street NW, Calgary, AB, T2N 2T9, Canada; 3 Rotman Institute of Philosophy, Department of Philosophy, University of Western Ontario, London, ON, N6A 5B8, Canada; 4 Department of Medicine, University of Western Ontario, 339 Windermere Road, London, ON, N6A 5A5, Canada; 5 Ottawa Hospital Research Institute, Clinical Epidemiology Program, Civic Campus, 1053 Carling Avenue, Ottawa, ON, K1Y 4E9, Canada; 6 Department of Medicine, Faculty of Medicine, University of Ottawa, Ottawa, ON, K1H 8L6, Canada; 7 Graduate School of Education and Statistics Department, Wharton School, University of Pennsylvania, 3700 Walnut Street, Philadelphia, PA, 19104, USA; 8 Department of Epidemiology and Community Medicine, University of Ottawa, Ottawa, ON, K1H 8M5, Canada; 9 Robarts Clinical Trials, Robarts Research Institute, London, ON, N6A 5K8, Canada; 10 Institute of Health and Society, Newcastle University, Baddiley-Clark Building, Richardson Road, Newcastle upon Tyne, NE2 4AX, UK; 11 Department of Medicine, University of Ottawa and Ottawa Hospital, Ottawa Hospital Research Institute, 1053 Carling Avenue, Ottawa, ON, K1Y 4E9, Canada; 12 Centre for Health Services Sciences, Sunnybrook Health Sciences Centre, 2075 Bayview Avenue, Toronto, ON, M4N 3M5, Canada

## Abstract

This article is part of a series of papers examining ethical issues in cluster randomized trials (CRTs) in health research. In the introductory paper in this series, we set out six areas of inquiry that must be addressed if the CRT is to be set on a firm ethical foundation. This paper addresses the first of the questions posed, namely, who is the research subject in a CRT in health research? The identification of human research subjects is logically prior to the application of protections as set out in research ethics and regulation. Aspects of CRT design, including the fact that in a single study the units of randomization, experimentation, and observation may differ, complicate the identification of human research subjects. But the proper identification of human research subjects is important if they are to be protected from harm and exploitation, and if research ethics committees are to review CRTs efficiently.

We examine the research ethics literature and international regulations to identify the core features of human research subjects, and then unify these features under a single, comprehensive definition of human research subject. We define a human research subject as any person whose interests may be compromised as a result of interventions in a research study. Individuals are only human research subjects in CRTs if: (1) they are directly intervened upon by investigators; (2) they interact with investigators; (3) they are deliberately intervened upon *via *a manipulation of their environment that may compromise their interests; or (4) their identifiable private information is used to generate data. Individuals who are indirectly affected by CRT study interventions, including patients of healthcare providers participating in knowledge translation CRTs, are not human research subjects unless at least one of these conditions is met.

## Introduction

This article is part of a series of papers examining ethical issues in cluster randomized trials (CRTs) in health research. CRTs are used increasingly in knowledge translation research, quality improvement research, community based intervention studies, public health research, and research in developing countries. While a small and growing literature explores ethical aspects of CRTs, cluster trials raise difficult issues that have not been adequately addressed. In the introductory paper in this series, we set out six areas of inquiry that must be addressed if the cluster trial is to be set on a firm ethical foundation [1]. These include identifying human research subjects, obtaining informed consent, the applicability of clinical equipoise, benefit-harm analysis, the protection of vulnerable populations, and the role and authority of gatekeepers in CRTs. This paper addresses the first of the questions posed, namely, who is the research subject in a CRT in health research?

CRTs are used in a range of fields, including education, criminology, public health, and health services research [2]. The identification of human research subjects must logically occur prior to the application of protections as set out in research ethics and regulation. Yet, the identification of human research subjects in a CRT may be unclear. Aspects of CRT design, including the fact that in a single study the units of randomization, experimentation, and observation may differ, complicate the identification of human research subjects. Are individual cluster members always human research subjects? In some CRTs, health professionals are randomly assigned to receive an experimental intervention, and their patients are the source of outcome data. Are the health care professionals human research subjects? Are patients human research subjects if the intervention administered to the healthcare professional indirectly affects their care?

The proper identification of human research subjects in a CRT is important for two reasons. First, misidentifying human research subjects in CRTs can have important consequences. If we fail to identify human research subjects in a CRT, then we may not be able to protect their interests adequately. If we are overly inclusive in identifying human research subjects, investigators will be subjected to regulatory burdens that may hamper important research. Second, as a pragmatic concern for research ethics committees, human research subjects must be correctly identified before ethical and regulatory requirements of informed consent, harm-benefit analysis, and fair subject selection can be applied. If research ethics committees are to review CRTs efficiently, human research subjects must be identified accurately.

This paper proposes a definition of human research subject that investigators and research ethics committees can apply to all CRTs in health research. We contend that the answer to the question "who is the research subject in a CRT in health research?" may vary, depending on the study design, population, or intervention under investigation. The definition proposed in this paper will help ensure that subjects in CRTs are identified appropriately and protected without needlessly hampering important research.

## Examples: Challenges in identifying the human research subject in CRTs

CRTs are heterogeneous with respect to design, population, and intervention. The following four examples illustrate the complexity of identifying human research subjects in CRTs and highlight the need for a definition of human research subject that can be employed across the spectrum of CRTs in health research.

### Example 1: The COMMIT trial

The Community Intervention Trial for Smoking Cessation (COMMIT) was designed to test the effectiveness of a comprehensive, community-oriented approach to influence citizens' smoking behaviours [3,4]. Twenty-two (22) communities with populations between 50,000 and 250,000 in the USA and Canada were paired on geographic location, size, and socio-demographic factors and one community in each pair was randomly assigned to the intervention. The intervention, which was delivered over a 4-year period, required active involvement of the communities and included activities focused on public education using mass media and organized community events, training of health care providers in cessation techniques, promotion of smoke-free policies in health care facilities and worksites, promotion of policies to restrict the sale of tobacco to youth, and development of smoking cessation resources and activities in each community. Population-based surveys, using random digit telephone dialing, were used to measure outcomes in the study. Prior to randomization of communities, and again at the end of the intervention, cross-sectional samples of approximately 2500 households per community were surveyed about their smoking behaviours. In addition, cohorts of approximately 550 heavy and 550 light-to-moderate smokers, who were willing to be contacted annually about their smoking status, were identified in each community. Main outcome measures were quit rates in the cohorts of smokers, as well as cross-sectional changes in the prevalence of smoking from pre- to post-intervention. Although the intervention significantly improved quit rates among light-to-moderate smokers, there was no significant impact on quit rates among heavy smokers or on the community prevalence of smoking.

Who were the human research subjects in this study? All residents in intervention and control communities? Only those residents participating in the telephone surveys? The cohorts of smokers followed prospectively over time?

### Example 2: A CRT of bed net distribution to reduce malaria prevalence

Sochantha and colleagues [5] report on a CRT designed to evaluate the impact of widespread distribution of insecticide-treated bed nets on malaria morbidity and mortality in remote areas of Cambodia. Thirty-six (36) villages, not previously included in the national malaria control program, were paired on their baseline prevalence of malaria and one village in each pair was randomly assigned to the intervention. Villages in the control arm received bed nets at the end of the study. Volunteers were identified in control and intervention villages and trained to recognize malaria symptoms, conduct diagnostic tests, and administer treatment. The volunteers conducted passive surveillance by recording information about the age and sex of the patient, the test results and treatments given. Ethical approval was obtained from Cambodia's Ministry of Health. The main outcomes were proxy measures of malaria incidence and the prevalence of malaria after the intervention. Malaria incidence was estimated using the number of positive consultations with malaria workers, with local population size obtained from census lists maintained by village heads. Malaria prevalence was estimated using blood samples collected from cross-sectional samples of approximately 250 randomly selected residents per village before and after the intervention. The intervention did not have a significant impact on malaria prevalence, but a non-significant trend towards decreased malaria incidence was observed.

Who were the human research subjects in this study? All residents in intervention and control villages? Those who received bed nets? Those who provided blood samples?

### Example 3: A CRT comparing interventions to improve primary care prescribing

Naughton and colleagues [6] report on a CRT designed to compare the effectiveness of two quality improvement interventions to increase prescribing of antiplatelet and lipid lowering medications for patients with cardiovascular disease or diabetes mellitus. A total of 110 General Practitioners (GPs) from 98 family practices in Ireland agreed to participate. Practices were randomly assigned to the intervention or control arm of the study. GPs in the intervention arm received a postal bulletin containing a personalized summary of their prescribing practices plus an educational outreach visit, while GPs in the control arm received a postal bulletin alone. Prescription data used to prepare the personalized summaries were obtained from the national pharmacy claims insurance database, which contains unique patient and GP identification numbers, as well as demographic information about the patients. The personalized summaries contained aggregate information about prescription rates and no information that could be used to identify individual patients. Ethical approval was obtained from the Irish College of General Practitioners. The main outcome measure was the change in the proportion of patients with cardiovascular disease or diabetes receiving appropriate drug therapies from pre- to post-intervention. All GPs received a postal survey questionnaire, which included questions about the perceived effects of the intervention on their practice. There were small increases in prescribing of preventive therapies in both arms of the study, but no significant differences between the arms.

In this study, there were no direct interventions on patients, nor use of identifiable private information. Who were the human research subjects in this study? All patients in participating practices? All of the cardiovascular disease and diabetes mellitus patients in participating practices? The physicians receiving the interventions [7,8]?

### Example 4: A CRT comparing modes of educating patients prior to breast cancer surgery

Goel and colleagues [9] report on a CRT designed to evaluate a decision aid to inform early breast cancer patients about their treatment options. General surgeons practicing in community hospitals in Ontario were invited to participate in the study. Interested surgeons were randomly assigned to either the intervention or control arm. Surgeons in both arms were asked to identify and enroll eligible patients presenting for breast biopsies. Surgeons in the intervention arm were directed to administer the decision aid (which consisted of an audiotape and a workbook), while surgeons in the control arm were directed to administer a simple educational pamphlet (which contained the same information as the workbook but no graphics or exercises). Of 232 surgeons invited to participate in the study, 76 expressed an interest, but only 57 surgeons ultimately enrolled patients into the trial (86 patients in the decision aid arm and 50 in the pamphlet arm). The study was approved by the Research Ethics Board at the University of Toronto. The main outcome measures were patient scores on a decisional conflict scale, breast cancer knowledge, anxiety, and decisional regret. Data were obtained from patient questionnaires completed at the initial consultation, prior to surgery, and 6 months after enrolment. Research nurses extracted data on pathology and actual treatment received from the patients' charts. No significant differences were observed between the study arms in knowledge, anxiety or regret, but a non-significant trend toward lower decisional conflict was observed in the decision aid group.

The intervention under study was directed at patients, but administered by their surgeons. Who were the human research subjects in this study? The patients? The surgeons?

## Methods

This paper seeks to provide a comprehensive definition of human research subject that is broadly applicable to CRTs in health research. The method used in this paper is one that philosophers call 'reflective equilibrium' [10]. The process of reflective equilibrium is meant to align a general rule that guides a person's reasoning on a particular issue -- in this case, about who is a human research subject -- and the particular judgments he or she holds on that issue. Using this method, we arrive at a definition that serves as the basis for reasoning about who is a human research subject. We then use both the process and the definition to reflect on new cases and develop a set of criteria for the identification of human research subjects.

Following the method of reflective equilibrium, we first review regulations and the research ethics literature for criteria that have been developed to identify human research subjects. Currently available regulatory definitions of research subjects are based on lists of procedures, i.e., an individual may only be classified as a research subject if he or she undergoes a procedure listed in the regulations [11-15]. These regulations constitute prior judgments that regulators have made about who is a research subject. We will employ these regulatory judgments as a foundation on which to construct a general definition of human research subject: we posit that a research subject is an individual whose interests are put at risk as a result of interventions in a research study. Thus, our definition of human research subject will capture considered convictions about who is -- and is not -- a research subject.

However, the method of reflective equilibrium is not simply a unidirectional distillation of a general definition from particular judgments about who is a human research subject. It requires that as we develop the definition, if we discover that any particular judgment we hold conflicts with it, we then have a decision to make about how to proceed: we may either revise the definition to accommodate the commitment, or we may rethink the judgment that is in tension with the definition developed thus far. Neither the general definition nor particular commitments are privileged over the other. This approach allows both reflection on whether or not current regulatory criteria that identify research subjects are adequate, as well as reflection on novel research designs, such as CRTs.

We use the method of reflective equilibrium to specifically develop criteria for the identification of human research subjects in CRTs. We examine whether the effects of group-level environmental interventions are, by themselves, sufficient to make cluster members human research subjects, and consider the impact of random assignment on an individual's status as a human research subject. We conclude by suggesting ways that our definition of human research subject may be applied in a variety of CRT designs and contexts to identify research subjects who are entitled to regulatory protections.

## Identifying human research subjects

### 1. Regulatory definitions of human research subject

The definition of research subject is a foundational question in the ethics of human subjects research. Nevertheless, many national and international research ethics guidelines offer no definition of human research subject [16-20]. One of the few national regulations that does so is the United States federal regulations on the "Protection of Human Subjects" [11]. Subpart A of these regulations is known as the Common Rule and governs all human subjects research conducted or funded by departments of the US federal government.

According to the Common Rule, a human research subject is a:

"living individual about whom an investigator (whether professional or student) conducting research obtains

(1) Data through intervention or interaction with the individual, or

(2) Identifiable private information.

*Intervention *includes both physical procedures by which data are gathered (for example, venipuncture) and manipulations of the subject or the subject's environment that are performed for research purposes. *Interaction *includes communication or interpersonal contact between investigator and subject. *Private information *includes information about behavior that occurs in a context in which an individual can reasonably expect that no observation or recording is taking place, and information which has been provided for specific purposes by an individual and which the individual can reasonably expect will not be made public (for example, a medical record). Private information must be individually identifiable (i.e., the identity of the subject is or may readily be ascertained by the investigator or associated with the information) in order for obtaining the information to constitute research involving human subjects [11]."

The Australian *National Statement on Ethical Conduct in Human Research *(hereafter, the "National Statement"), meanwhile, lists six types of activities that confer human research subject status on an individual, including:

"1. taking part in surveys, interviews, or focus groups;

2. undergoing psychological, physiological or medical testing or treatment;

3. being observed by researchers;

4. researchers having access to their personal documents or other materials;

5. the collection and use of their body organs, tissues or fluids (e.g., skin, blood, urine, saliva, hair, bones, tumour and other biopsy specimens) or their exhaled breath;

6. access to their information (in individually identifiable, re-identifiable, or non-identifiable form) as part of an existing published or unpublished source or database" [12].

Four of these criteria (1, 2, 3, and 5) describe different kinds of interventions upon or interactions with subjects, and are therefore reducible to the first item in the Common Rule criteria ("intervention or interaction with the individual"). The fourth and sixth items in the Australian National Statement criteria refer to the use of an individual's information, including both identifiable private information and information from which personal identifiers have been removed [12].

Because of their comprehensiveness, and because they are supported by a great deal of historical documentation, we will use the Common Rule criteria as our starting point. We will draw on other guidelines, such as the Australian National Statement, where applicable. We now ask, do the criteria of the Common Rule share a core feature? If one can be identified, it may provide the basis for a comprehensive, generalizable definition of human research subject.

### 2. Distinctive features of human research subjects

In attempting to identify a core feature linking the criteria outlined in the Common Rule and other regulations, we are guided by normative work on the distinction between a patient in clinical practice and a human subject in clinical research. In the appendices to the *Belmont Report*, Robert Levine draws a distinction between medical practice and clinical research based on the purpose of each activity [21]. Clinical practice, he argues, involves a physician acting solely for the purpose of ameliorating the health of the patient. Research, on the other hand, is "...a systematic investigation, including research development, testing and evaluation, designed to develop or contribute to generalizable knowledge" [11]. Research may include interventions that offer benefit to research subjects, but these are not essential components of an activity whose primary purpose is to benefit society and generate knowledge. The difference between the purpose of clinical practice and that of clinical research leads to an important distinction between the physician-patient and investigator-subject relationship.

In clinical practice, the physician and patient have a fiduciary relationship. A fiduciary relationship is a relationship of structural inequality in which the beneficiary (in this case, the patient) entrusts the fiduciary (in this case, the physician) with discretionary power over important practical interests (in this case, the medical interests of the patient) [22]. As a result of ceding power to the fiduciary, the beneficiary is dependent on the fiduciary and vulnerable to harm. Accordingly, a number of duties accrue to the fiduciary, including a duty to act and advise so as to protect the interests at stake. In the case of the physician-patient relationship, physicians are obligated to protect and promote the medical interests of their patients, and to protect their privacy. Levine points out that in ordinary clinical practice, patients can be confident that physicians will endeavor to protect these interests [21].

The relationship between researchers and human research subjects is somewhat different. Levine and Rothman note that physician-researchers have conflicting obligations [21,23]. On the one hand, they have obligations to protect the interests of their patient-subjects [21-23]. On the other hand, physician-researchers also have obligations to the study, such as ensuring compliance with experimental treatment protocols. These may conflict with their obligations to protect patient welfare [21,23]. As Rothman writes, "The bedrock principle of medical ethics -- that the physician acted only to promote the well-being of the patient -- did not hold in the laboratory...The doctor-patient relationship could no longer serve as the model for the investigator-subject relationship." [23]

Human research subjects are vulnerable because a physician-investigator's obligation to protect subjects may conflict with scientific obligations. As Levine puts it, the role of a research subject approximates that of a means to an end [21]. Both the Common Rule and Australian National Statement specify ways in which a subject's interests may be compromised for scientific purposes. When a researcher intervenes on a human research subject, either with an experimental intervention or to collect data, the research subject's welfare may be at risk. The same is true when a researcher interacts with a research subject. By collecting personal information, the researcher may violate the subject's privacy.

### 3. A definition of human research subject

As noted above, regulatory criteria for human research subjects may be understood as specifying ways in which an individual's interests may be compromised for scientific purposes. Based on this finding, we propose the following definition of a research subject:

A research subject is an individual whose interests may be compromised as a result of interventions in a research study.

Interventions (using the term in its broadest sense) refer to the procedures under investigation in both the experimental and control arms of the study, as well as non-therapeutic data collection procedures. Interests refer to the goods that an individual would ordinarily seek to protect, including health, welfare, and privacy.

We find historical support for this definition in the 1974 US Department of Health, Education, and Welfare (DHEW) regulations for human subjects research -- the immediate precursor to the Common Rule [24]. Rather than define human research subject *per se*, the DHEW regulations refer to "subjects at risk". A subject at risk is:

"any individual who may be exposed to the possibility of injury, including physical, psychological, or social injury as a consequence of participation as a subject in any research, development, or related activity which departs from the application of those established and accepted methods necessary to meet [the individual's] needs, or which increases the ordinary risks of daily life, including the recognized risks inherent in a chosen occupation or field of service." [24]

The authors of the DHEW regulations identify effectively the same core feature of human research subject as we do, namely, "the possibility of injury, including physical, psychological, or social injury as a consequence of [research] participation" [24].

### 4. Evaluating current regulations with the definition

Our novel definition of human research subject may be used to critically evaluate regulatory criteria for human research subjects. We use this process to identify our own set of criteria for the identification of human research subjects. As previously noted, the Australian National Statement offers a six-item list of ways in which an individual may become a human research subject. Four of these criteria (1, 2, 3, and 5) identify ways in which researchers may intervene upon or interact with subjects. These items are, therefore, consistent with our definition of human research subject. The fourth criterion refers to "researchers having access to personal documents or materials." Such access may compromise subjects' privacy; this criterion, therefore, merits inclusion [12]. The sixth criterion defines any individual from whom a researcher obtains "information (in individually identifiable, re-identifiable, or non-identifiable form) as part of an existing published or unpublished database or source" as a human research subject [12]. While the use of identifiable (and re-identifiable) information may present a risk to an individual's privacy, the use of non-identifiable information presents no such risk. We conclude that the use of non-identifiable information should not be included in criteria identifying human research subjects.

Are the components of the Common Rule consistent with our definition of human research subject? The Common Rule criteria include both interventions on and interactions with subjects, as well as the use of identifiable private information. These constitute means by which research subjects' interests may be compromised, and, therefore, merit inclusion in criteria specifying human research subjects (see Table 1). The Common Rule widens the description of interventions to include both "physical procedures" and "manipulations of the subject or the subject's environment." [11] Given that physical procedures or manipulations of the subject necessarily involve some degree of risk, and may compromise the subject's welfare interests, they merit inclusion. However, the relevance of "manipulations...of the subject's environment" to human research subjects requires detailed examination.

**Table 1:** The identification of human research subjects: a definition and a set of criteria.

Definition	A human research subject is an individual whose interests may be compromised as a result of interventions in a research study.
**Criteria**	A human research subject is:
	1. An individual who is directly intervened upon by an investigator;
	2. An individual who is deliberately intervened upon via manipulation of the individual's environment by an investigator;
	3. An individual with whom an investigator interacts for the purpose of collecting data; or,
	4. An individual about whom an investigator obtains identifiable private information for the purpose of collecting data.

## The importance of environmental manipulation

As specified in the Common Rule, one way in which an investigator may intervene on human research subjects is by manipulating their environment. With respect to healthcare CRTs, Mann and Reyes have interpreted this to mean that an intervention designed to alter a healthcare professional's practice pattern entails a manipulation of the environment of all patients whose care may be affected [7]. According to Mann and Reyes, every patient of a professional whose care may be influenced by a study intervention in a CRT meets the regulatory definition of a human research subject. This claim is understandable, given that much of the literature on the ethics of CRTs assumes that all cluster members (in this case, patients) will be subjects [2,20,25-28]. If correct, this view has important implications for the conduct of CRTs. If, in CRTs targeted at health professionals or health systems in general, all patients are considered human research subjects, the administrative burdens associated with protecting patients as human research subjects may threaten the feasibility of trials. We explore below whether the indirect effects of a research intervention at the group level (such as an educational intervention administered to health professionals) implies that all individuals within the group (including the patients of professionals participating) must be considered human research subjects.

### 1. Does environmental manipulation confer the status of human research subject on individuals?

The CIOMS *International Ethical Guidelines for Biomedical Research Involving Human Subjects *defines research as any study that manipulates an individual's social or physical environment [17]. These guidelines, together with the CIOMS *International Ethical Guidelines for Epidemiological Studies*, address issues of informed consent and harm-benefit analysis in epidemiologic research [20]. Therefore, without actually defining research subject, both CIOMS guidelines appear to acknowledge that individuals who may be directly affected by public health interventions that manipulate the environment, such as water fluoridation or pesticide use, are human research subjects [17,20].

An unduly broad interpretation of environmental manipulation is, however, untenable. It seems implausible that everyone whose environment is manipulated in the context of a research project should be considered a human research subject. The term environment refers to "the surroundings or conditions in which a person, animal, or plant lives or operates" [29]. Using this definition, "manipulations of the...subject's environment" would imply that entire populations might be involved in a research study. For example, studies in particle physics at the Large Hadron Collider meet the Common Rule definition of research, in that they are systematic investigations designed to develop generalizable knowledge and constitute "manipulations of...the environment" [11]. Do the citizens of France and Switzerland thus become human research subjects? If experiments at the Large Hadron Collider are theoretically capable of producing microscopic black holes, does that imply that everyone on earth is a human research subject [30]? Clearly, a more restrictive interpretation of environmental manipulation is required.

It is helpful to consider what the US National Commission for the Protection of Human Subjects of Biomedical and Behavioral Research meant when it included environmental manipulation in its definition of a human research subject. In a paper prepared for the National Commission in 1975 (included in the 1979 appendix to the *Belmont Report*), Robert Levine defined research as "...manipulation, observation, or other study of a human being -- or of anything related to that human being that might subsequently result in manipulation of that human being -- done with the intent of developing new knowledge and which differs in any way from customary medical (or other professional) practice" [22]. Specific reference to manipulation of an individual's environment did not appear until the National Commission's 1978 *Report and Recommendations: Institutional Review Boards *which replaced Levine's definition of "anything related to that human being that might subsequently result in manipulation of that human being" with "manipulations of...the subject's environment" [31]. This latter language was subsequently incorporated into the Common Rule. Exploring the reasons for this change in regulatory language helps us understand how to interpret environmental manipulation in the context of human subjects research.

Given the research ethics literature at the time, it seems likely that the National Commission was seeking to protect individuals who participated in studies evaluating the psychological effects of various environmental stimuli. Inclusion of environmental manipulation in the criteria for human research subjects seemed intended to cover research that deliberately manipulated subjects and jeopardized their welfare without physical contact. Examples of such research include studies examining the psychological and behavioural effects of habitation in simulated fallout shelters sponsored by the US Office of Civil and Defense Mobilization and studies evaluating the psychological effects of other environmental manipulations, such as sensory deprivation [32-34]. Environmental manipulations in the Civil Defense studies involved living in a confined space for prolonged periods of time, crowding, and variations in air quality, temperature and the availability of potable water. These manipulations of the environment caused human research subjects physical and psychological discomfort and jeopardized their welfare.

These studies, and the ethical concerns that they highlight, support a narrow reading of the environmental manipulation clause. We suggest that environmental manipulation must be designed to have a direct effect on individuals if they are to be considered human research subjects. We believe that this interpretation is consistent with the intent of the National Commission. We will therefore expand our criteria specifying human research subjects to include individuals who are deliberately affected *via *manipulation of their environment (see Table 1).

Our conclusion is also consistent with the language enshrined in the final report of the National Bioethics Advisory Commission. In its 2001 *Report and Recommendations: Ethical and Policy Issues in Research Involving Human Participants*, the Commission agreed that the term human research subject "connotes the fact that the individual is 'subjected' to an action by the investigator." [35] The Commission specifically recommended that "Research be considered to involve human participants when individuals 1) are exposed to manipulations, interventions, observations or other interactions with investigators or 2) are identifiable through research using biological materials, medical and or other records, or databases" [35].

### 2. Do indirect effects of CRT interventions on health professionals or health systems confer the status of human research subject on patients?

Mann and Reyes assert that educational or quality improvement interventions in a CRT designed to change physicians' practice patterns constitute manipulations of the environment of participating physicians' patients [7]. As a result, they conclude that all patients of physicians whose care may be indirectly affected in such trials are human research subjects. We respectfully disagree.

First, for an individual to be properly considered a human research subject, the environmental manipulation must be designed to produce a direct effect on that individual. This is not the case in CRTs that intervene on health professionals. The interventions under evaluation in these studies are intended to change health professionals' behaviour by increasing their use of evidence-based strategies to improve care. Patients are not manipulated by these interventions; they are only indirectly affected by them.

Second, even if a change in professionals' practice patterns did constitute a deliberate manipulation of their patients' environment (a claim that we do not grant), that manipulation does not jeopardize patients' interests or mean that they ought to be considered human research subjects. Distinguishing between clinical practice and clinical research may help clarify matters, As Levine writes:

"If a physician proceeds in [the] interaction with a patient to bring what [the physician] considers to be the best available technique and technology to bear on the problems of that patient with the intent of doing the most possible good for that patient, this may be considered the pure practice of medicine. By contrast, if a physician interacts with an individual with the intent of developing new knowledge (not primarily for the benefit of that individual), this activity may be classified as research" [21].

In a CRT that intervenes on health professionals, the participating health professionals are research subjects, not investigators. They are still bound by professional responsibility to act in their patients' best interests. Thus, patient welfare is not put at risk in CRTs that evaluate interventions aimed at improving practice.

Some studies evaluate patient level effects as an outcome measure. The fact that a patient level effect may be measurable is relevant to patients only insofar as their private health information may be used, or they may be asked to submit to surveys or additional examinations to evaluate the outcome of the CRT. When this occurs, the patients of health professionals participating in an educational or quality improvement CRT ought to be considered human research subjects.

CRTs evaluating quality improvement initiatives or system-wide innovations aimed at healthcare organizations should be treated similarly to CRTs evaluating educational interventions aimed at providers. Patients need only be considered research subjects if they are directly intervened upon, if they interact with investigators, or if identifiable private information is used.

CRTs evaluating novel modes of healthcare delivery must be treated differently if the experimental intervention involves a departure from standard care [36]. Such CRTs are conducted because the efficacy of the mode of delivery is uncertain. Randomization to clusters is undertaken for logistical reasons and to avoid treatment contamination. Novel modes of healthcare delivery are therefore best thought of as experimental interventions directed at patients. In such studies, the patients should be considered research subjects because they are directly intervened upon.

### 3. Implications for CRTs in fields other than healthcare

In some CRTs, particularly in public health, the purpose of the experimental intervention is to deliberately manipulate individuals via their environment. For example, in the COMMIT study, described above, multi-modal community interventions (environmental manipulations) were intended to produce behavioral change in smokers living in intervention communities [3,4]. Another CRT evaluating interventions aimed at individuals via environmental manipulation compared rates of diarrheal illness in communities randomly assigned to water treatment with flocculant disinfectant or a control [37]. In these studies, the purpose of the environmental manipulation is to intervene on individual community members. The residents of communities in these studies are therefore human research subjects and are entitled to regulatory protections. However, this does not mean that informed consent is required. Rather, many of these studies would meet criteria for a waiver of informed consent that are laid out in various national and international research ethics guidelines [11,17,18,20]. These guidelines generally require that the research not be feasible if consent were required, and that the interventions in the study pose only minimal risk to subjects -- conditions that are often met by CRTs in public health. (When informed consent must be obtained in CRTs is the subject of another paper in this series).

CRTs in education are roughly analogous to CRTs in healthcare and the teacher-student relationship has many of the characteristics of a fiduciary relationship [21]. If a CRT is used to evaluate the effect of a continuing education intervention for teachers, the indirect effect on students does not require treating them as human research subjects. However, CRTs of experimental curricular programs may be analogous to CRTs evaluating novel methods of health service delivery, and may require treating students as human research subjects.

## What is the importance of random intervention assignment?

The ethical implications of random assignment to study interventions in CRTs are unclear. In CRTs, random group assignment often takes place before subjects are enrolled [2,25,28]. In CRTs of large cluster level public health interventions, clusters may be randomly assigned to interventions that some cluster members never receive (e.g., smoking cessation messages are not necessarily received by all community members). As the literature to date has broadly assumed that all cluster members are human research subjects, this has led to the conjecture that the act of random assignment may in itself be sufficient to confer the status of human research subject on cluster members [28]. In randomized controlled trials (RCTs), assignment of individuals to the intervention or control arm is determined by a mechanism beyond the control of the individual; it is not determined by the physician-patient dyad. Random assignment has thus been viewed as a research intervention [38]. The corollary of this is that randomization of individuals or groups may confer the status of human research subject upon those randomized.

We argue that random assignment does not confer the status of human research subject. Whether or not it is random, intervention assignment is beyond the control of the individual or group. For this reason, the use of random intervention assignment is immaterial to the determination of whether an individual is a research subject. If some non-random method of intervention assignment were used in place of random assignment, the threats to subjects' liberty and welfare interests would be unchanged. A human research subject's interests are jeopardized by participating in research--whether intervention assignment is random or otherwise. Random assignment of clusters ought not be considered a criterion for the identification of human research subjects. The fact that, in many CRTs, group assignment is determined before subject enrolment should be acknowledged in consent discussions with individuals who are identified as human research subjects.

## Implications for cluster randomized trials

Using a novel definition of human research subject, we have set out four criteria for their identification (see Table 1). Here we explore the implications of each of these criteria for CRTs in health research.

### Criterion 1. An individual who is directly intervened upon by an investigator for research purposes

Individuals ought to be considered human research subjects if, in the context of a research study, they are the recipients of a study intervention (active or control) or if they undergo an intervention to collect data, such as venipuncture.

If an intervention is targeted at individuals but random assignment takes place at the cluster level (typically to avoid treatment contamination or for logistical reasons) then the individuals receiving the intervention should be considered human research subjects. In healthcare, this includes CRTs evaluating therapeutic or health promotion modalities aimed at individual patients, as well as CRTs evaluating new modes of health service delivery. An example of the former is a CRT evaluating the effect of individualized exercise prescriptions for patients randomized by physician practice [39]. An example of the latter is a CRT evaluating the effectiveness of asthma management using specialist nurses [36]. In these studies, the individual patients themselves are being intervened upon and should therefore be considered human research subjects.

In many healthcare CRTs, the intervention under study is not administered to patients, but rather to healthcare professionals, even though outcomes are evaluated using patient data. It may be asked whether health professionals who receive an educational intervention in a CRT are human research subjects or collaborators. Collaborators are individuals who contribute to the design of or participate in the conduct of a research study. They are not recipients of study interventions. In knowledge translation CRTs, the health professionals are receiving an experimental educational or quality improvement intervention. When they are directly intervened upon in this way, health professionals are human research subjects [7,8,40].

Some healthcare CRTs evaluate complex interventions that may include a combination of health professional education, novel modes of health service delivery, and patient level interventions [41]. In these cases, determining whether healthcare providers or patients or both ought to be considered human research subjects requires that research ethics committees trace the impact of each study intervention and data collection procedure. When study interventions are directed at health professionals, they are human research subjects. Patients ought to be considered research subjects if: patient level interventions (either therapeutic interventions or data collection procedures) or novel modes of health service delivery are used; researchers interact with them; or, the study uses their identifiable private information to evaluate outcomes.

### Criterion 2. An individual who is deliberately intervened upon via manipulation of the individual's environment by an investigator for research purposes

Individuals who are intervened upon through manipulation of their environment ought to be considered human research subjects. This includes individuals affected by public health interventions in CRTs, whether the unit of randomization is a municipality, a neighbourhood, a family, or some other group. Because these individuals are human research subjects, they are entitled to regulatory protections, including assessment by a research ethics committee of study benefits and harms. These studies may meet regulatory criteria for a waiver of informed consent if interventions pose minimal risk and the study is not feasible if individual informed consent must be obtained.

We concluded above that the indirect effects of a study intervention on an individual are not sufficient to warrant considering that person a human research subject. In healthcare CRTs, patients may be indirectly affected by educational or quality improvement interventions that are directed at healthcare professionals or institutions. The physicians under study continue to have an obligation to act in their patients' best interests, and have no competing obligations to the study itself. Thus, the physician-patient fiduciary relationship is preserved. If there are no patient level interventions, the researcher has no interaction with individual patients, and there is no use of identifiable private information for research purposes, patients are not human research subjects.

### Criterion 3. An individual with whom an investigator interacts for the purpose of collecting data

An individual from whom an investigator, in the context of a research study, obtains data through interaction should be considered a human research subject. Interaction includes communication or interpersonal contact between investigator and subject, for example, interviews, focus groups, or questionnaires. Data collection through interaction entails that respondents are entitled to protections as human research subjects [11,12].

### Criterion 4. An individual from whom an investigator obtains identifiable private information for the purpose of collecting data

Obtaining identifiable private information about people within a cluster means they ought to be considered human research subjects, and that they are entitled to protection. Conversely, there is no risk to an individual's privacy if the researchers are only collecting anonymized or aggregate group-level information [42]. Individuals whose data have been anonymized before transfer to the investigators, or whose administrative or health-related information is used to generate aggregate measures for a cluster are not human research subjects unless they are intervened upon in some other way.

## Practical applications for ethics review of CRTs

We will now apply our novel definition and criteria for the identification of human research subjects to the example CRTs.

### Example 1: The COMMIT Trial

The multi-modal community intervention constitutes an environmental manipulation directed at all community residents. Therefore, people in the intervention communities ought to be considered human research subjects. Data were collected using surveys of cross sectional samples of the community and prospective cohorts of smokers, and survey respondents (whether from intervention or control communities) are human research subjects. Residents of control communities were not intervened upon, did not interact with investigators (unless they completed surveys), and did not contribute identifiable private information. Therefore, residents of control communities who were not survey respondents are not human research subjects.

### Example 2: A CRT of bed net distribution to reduce malaria prevalence

The distribution of bed nets constitutes a direct intervention on individuals. Therefore, all residents of the intervention communities who received a bed net ought to be considered human research subjects. Individuals contributing blood samples, whether from intervention or control communities, were directly intervened upon and are therefore human research subjects. Members of control communities were not intervened upon (unless they contributed blood samples), did not interact with investigators, and did not contribute identifiable personal information. Therefore, members of control communities who did not contribute blood samples are not human research subjects.

### Example 3: A CRT comparing interventions to improve primary care prescribing

The physicians in this study were recipients of an experimental intervention in that they received one of two candidate quality improvement interventions, and ought to be considered human research subjects. The patients were indirectly affected by the interventions directed at the physicians, but they received no intervention from study personnel, had no interaction with study personnel, and contributed no identifiable private information. Therefore, the patients of physicians participating in this study are not human research subjects.

### Example 4: A CRT comparing modes of educating patients prior to breast cancer surgery

In this study, the patients were recipients of an experimental intervention in that they received one of two candidate modes of education about their surgical options. They responded to questionnaires to generate outcome data; they also contributed identifiable medical information. As a result, the patients ought to be considered human research subjects. The surgeons merely delivered the study interventions to the patients and were not themselves the recipients of interventions; they did not otherwise interact with researchers to provide data, nor did they provide identifiable private information. Thus, the surgeons are not human research subjects.

## Conclusions

We have defined a human research subject as an individual whose interests may be compromised as a result of interventions in a research study, and have specified four criteria for the identification of human research subjects (see Table 1). Human research subjects are: those intervened upon by researchers, either directly or by deliberate manipulation of their environment; those who interact with researchers to provide data; or those who provide identifiable private information.

In articulating a novel definition of a human research subject and a set of criteria for their identification, this paper represents an essential first step in addressing additional questions on how to protect human research subjects in CRTs. Subsequent papers in this series will use this definition of human research subject and address informed consent, clinical equipoise, the analysis of harms and benefits, subject selection, the protection of vulnerable subjects, and the role and authority of gatekeepers in CRTs in health research.

## Note

We have created a Wiki webpage to facilitate an open discussion about the ideas expressed in this and other papers published in the series on ethical challenges in CRTs. Please enter your thoughts and comments at http://crtethics.wikispaces.com.

## Competing interests

AB, JCB, ADM, RS, MT, CW, AW: None declared.

RB, AD, MPE, JMG, and MZ have all submitted cluster trial protocols to ethics committees and had difficulty explaining to them the differences between cluster randomized trials and individual patient clinical trials.

## Authors' contributions

ADM, CW, AB, AW, JMG, and MT contributed to the conception and design of the manuscript.

ADM wrote the initial draft and ADM and CW led writing of subsequent versions.

All authors commented on sequential drafts and approved the final version.
